# Supplementary material for: Genome-Wide Identification of CsATGs in Tea Plant and the Involvement of CsATG8e in Nitrogen Utilization
Source: Int J Mol Sci. 2020 Sep 24;21(19):7043. doi: 10.3390/ijms21197043 (PMC7583067; doi:10.3390/ijms21197043)
Supplement: Supplementary file 1 [file ijms-21-07043-s001.zip › Supplmentary Figure.pdf]

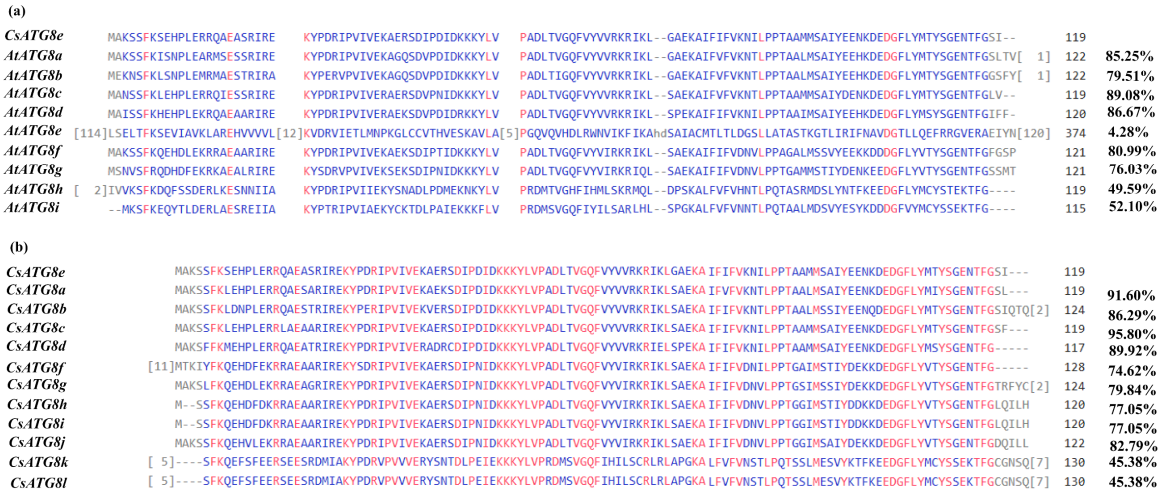

Figure S1. Amino acid sequence alignment of *CsATG8e* and ATG8 members in *Arabidopsis* and tea plant. (**a**) Comparison between *CsATG8e* and ATG8 members in *Arabidopsis*. (**b**) Comparison between *CsATG8e* and ATG8 members in tea plant. The number on the right is the identity with *CsATG8e* of the corresponding gene on the left side in one role.

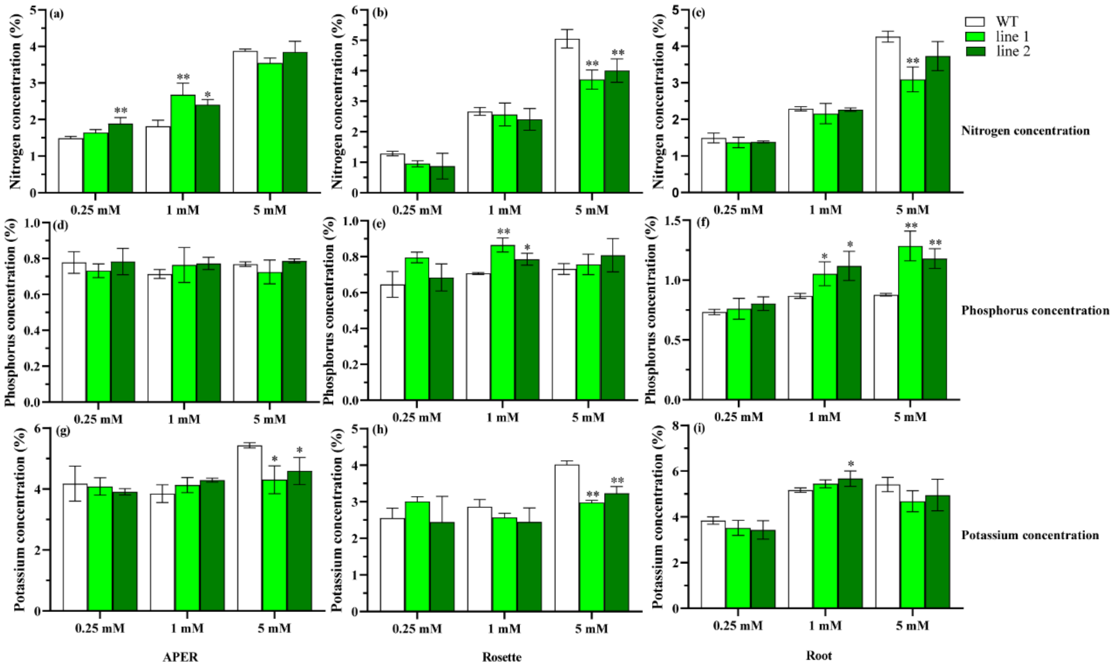

Figure S2. The concentration of N, P and K in overexpression *Arabidopsis* (OE) and wild type (WT) under three N regimes. (**a–c**) N concentration in aerial part excluding rosette (APER), rosettes (Rosette) and roots (Root) of OE and WT under 0.25 mM, 1 mM and 5 mM N treatments. (**d–f**) P concentration for APER, Rosette, and Root of OE and WT under 0.25 mM, 1 mM and 5 mM N treatments. (**g–i**) K concentration for APER, Rosette and Root of OE and WT under 0.25 mM, 1 mM and 5 mM N treatments. 0.25 mM N, 0.125 mM NH_4NO_3; 1 mM N, 0.50 mM NH_4NO_3; 5 mM N, 2.50 mM NH_4NO_3. Values are means ± standard (SD) (n = 4). Asterisks indicate significant difference between WT and two OE lines (line1, line2). * $p < 0.05$; ** $p < 0.01$ (t-test).

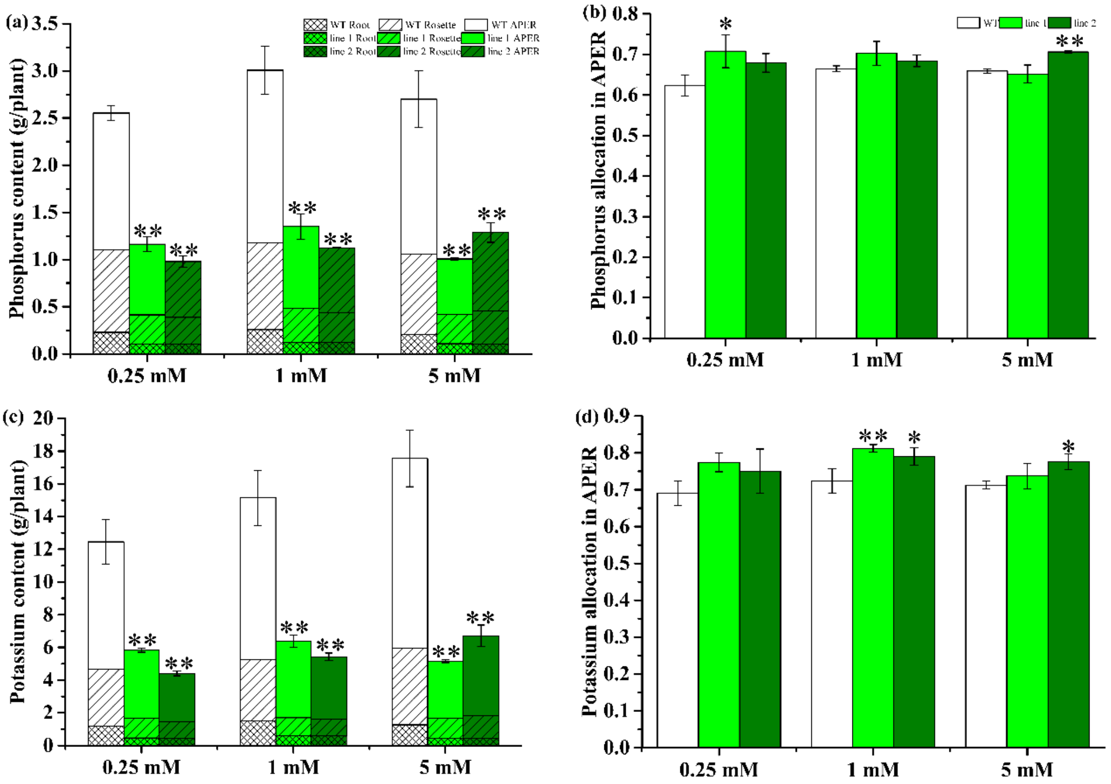

Figure S3. Analysis of P and K content and allocation in overexpression *Arabidopsis* (OE) and wild type (WT). (**a**) and (**c**) The composition of P and K in OE lines and WT. (**b**) and (**d**) The N and K allocation in aerial part excluding rosette (APER). Values are means ± standard (SD) ($n = 4$). Asterisks indicate significant difference between WT and two OE lines (line1, line2). * $p < 0.05$; ** $p < 0.01$ (t-test).
